# Neural stem cells from protein tyrosine phosphatase sigma knockout mice generate an altered neuronal phenotype in culture

**DOI:** 10.1186/1471-2202-7-50

**Published:** 2006-06-19

**Authors:** David L Kirkham, Laura KK Pacey, Michelle M Axford, Roberta Siu, Daniela Rotin, Laurie C Doering

**Affiliations:** 1 Department of Pathology and Molecular Medicine, McMaster University, Hamilton Ontario, L8N 3Z5, Canada; 2 Cell Biology Program, The Hospital for Sick Children and Department of Biochemistry, University of Toronto, Toronto, Ontario, M5G 1X8, Canada

## Abstract

**Background:**

The LAR family Protein Tyrosine Phosphatase sigma (PTPσ) has been implicated in neuroendocrine and neuronal development, and shows strong expression in specific regions within the CNS, including the subventricular zone (SVZ). We established neural stem cell cultures, grown as neurospheres, from the SVZ of PTPσ knockout mice and sibling controls to determine if PTPσ influences the generation and the phenotype of the neuronal, astrocyte and oligodendrocyte cell lineages.

**Results:**

The neurospheres from the knockout mice acquired heterogeneous developmental characteristics and they showed similar morphological characteristics to the age matched siblings. Although *Ptprs *expression decreases as a function of developmental age *in vivo*, it remains high with the continual renewal and passage of the neurospheres. Stem cells, progenitors and differentiated neurons, astrocytes and oligodendrocytes all express the gene. While no apparent differences were observed in developing neurospheres or in the astrocytes and oligodendrocytes from the PTPσ knockout mice, the neuronal migration patterns and neurites were altered when studied in culture. In particular, neurons migrated farther from the neurosphere centers and the neurite outgrowth exceeded the length of the neuronal processes from age matched sibling controls.

**Conclusion:**

Our results imply a specific role for PTPσ in the neuronal lineage, particularly in the form of inhibitory influences on neurite outgrowth, and demonstrate a role for tyrosine phosphatases in neuronal stem cell differentiation.

## Background

Neural stem cells comprise a relatively quiescent, uncommitted and multipotent subpopulation in the central nervous system [1-3]. Stem cells reside in multiple areas of the central nervous system including the olfactory bulb, striatum, cerebellum, hippocampus (dentate gyrus), cerebral cortex and spinal cord [4]. The subventricular zone (SVZ) is one of the richest zones capable of generating stem cells during development and into old age [5]. In culture, neural stem cells and progenitor cells grow as spherical cell clusters termed neurospheres, and they provide a valuable way to investigate neuronal and glial cell lineage development in vitro. Neural stem cells express EGF and FGF receptors [6] and the mitogens FGF and EGF are used to induce mitosis in stem cells [7,8].

During development, cell signaling facilitates cellular maturation as well as extracellular interactions between the cells and the environment [9]. Kinases and phosphatases are regulatory proteins antagonistically controlling the phosphorylation state of molecules within cell-signaling pathways. It had been previously demonstrated that protein tyrosine phosphatases (PTPs) contribute to neuronal development, influencing axon growth, trajectory, guidance, fasciculation and synapse formation [10-14]. Receptor protein tyrosine phosphatase sigma (PTPσ, also known as LAR-PTP2, PTP-P1, PTP-NU3, PTP-NE3, CRYPα and CPTP1), the product of the *Ptprs *gene, is a member of the LAR family of protein tyrosine phosphatases (Type IIa PTP) along with LAR and PTPδ [15]. It is comprised of a cell adhesion-like ectodomain consisting of 3 Ig and 5 or 8 FNIII repeats, a single transmembrane domain and 2 tandem catalytic domains, the first of which is active [16,17]. The ectodomain of PTPσ interacts with the heparan sulfate proteoglycans(HSPGs) agrin and collagen XVIII in the retina [18], although it has other, yet unidentified ligands in the muscle [18] and other tissues. Intracellular substrate(s) for PTPσ have not yet been described.

Using PTPσ knockout mice our previous studies and those of others have focused on the spatial patterns of PTPσ expression particularly in the central and peripheral nervous system, as well as on nerve regeneration and axonal guidance [20-24]. PTPσ deficient mice exhibit neuroendocrine defects, with reduced production of growth hormone and prolactin by the pituitary, which contribute to their high rate of postnatal mortality [21,25]. Surviving mice exhibit neuronal and neurobehavioral defects [20], and abnormal nerve regeneration pattern following injury of the sciatic [23] or facial nerves [24]. In addition, our analysis of the CNS revealed hippocampal dysgenesis, reduction in thickness of the corpus callosum and cerebral cortex and spinal cord abnormalities [22].

In our previous studies [22] we observed strong expression of PTPσ immediately adjacent to the ventricles in the subventricular zone (SVZ). Prompted by this observation we sought to determine if neural stem cells express the Ptprs gene and if this gene influenced the phenotype of the 3 principal lineages generated from these stem cells: astrocytes, oligodendrocytes and neurons. Our results demonstrate expression of PTPσ in stem cell-derived astrocytes, oligodendrocytes and neurons. Moreover, while differentiated astrocytes and oligodendrocytes from the neurospheres of PTPσ knockout mice appeared normal, the neurons showed an accelerated growth of processes, suggesting that PTPσ provides inhibitory signal(s) that is specific for the neuronal lineage.

## Results

### PTPσ does not affect neurosphere ontogeny and morphology

**Figure 1:**
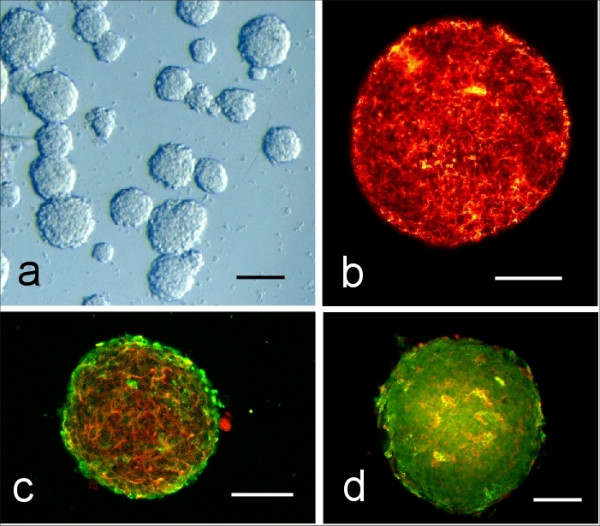
Neurospheres derived from the PTPσ(-/-) knockout mouse brain show normal morphological features. a) Classical appearance of neurospheres in culture. Clonal derived spheres from a PTPσ(-/-) mouse visualized by differential interference contrast microscopy. Scale bar = 50 μm b) Nestin expression in a medium sized neurosphere from a PTPσ(-/-) mouse. This stem cell/progenitor cell marker is uniformly distributed throughout the sphere. Confocal microscopy. Scale bar = 50 μm c) Double labeling for nestin (rhodamine-red) and the EGF-R (fluorescein-green) in a sphere derived from a PTPσ(+/-) mouse. Confocal microscopy. Scale bar = 50 μm d) Immunofluorescent image of the NG2 progenitor cell marker (fluorescein-green) and the neuronal marker tubulin (rhodamine-red) in a neurosphere generated from a PTPσ (-/-) mouse. Epi-fluorescence microscopy. Scale bar = 50 μm.

To determine the morphology of neurospheres generated from the PTPσ (-/-), (+/-) and (+/+) mice, we recovered stem cells from the SVZ of each genotype and generated neurospheres in the presence of bFGF and EGF. The property of self-renewal was demonstrated by the weekly passage of the spheres for several months and in some cases beyond one year. With the continued passage of the neurospheres, we were able to analyze several hundred neurospheres for each genotype. Neurospheres examined by phase contrast microscopy were indistinguishable between the 3 groups (genotypes) of mice. We established the neurosphere cultures at least 10 times from each genotype. While combinations of bFGF and EGF generated higher numbers of neurospheres in contrast to a single growth factor, there was no difference between the three genotypes (Table 1). As the neurospheres increased in size, many acquired a heterogenous cellular composition. The spheres contained cells with the progenitor cell markers nestin and NG2 (Figure 1). We observed a wide variety of markers expressed by cells in the neurospheres. Individual neurospheres would commonly reveal the co-expression of two lineages (Table 2). Many cells were stained for 2 markers in addition to the generation of multiple lineages. We could localize cell markers that labeled different cell populations in the spheres as well as cells co-expressing different lineage markers (Figure 1). Heterogeneity was most apparent in the largest spheres. There was no difference between the 3 genotypes regarding the single expression or co-expression of the various markers. In addition, the larger spheres also consisted of differentiated cells predominantly of a neuronal phenotype (Figure 1).

### Neural stem cells and differentiated progeny express PTPσ

**Figure 2:**
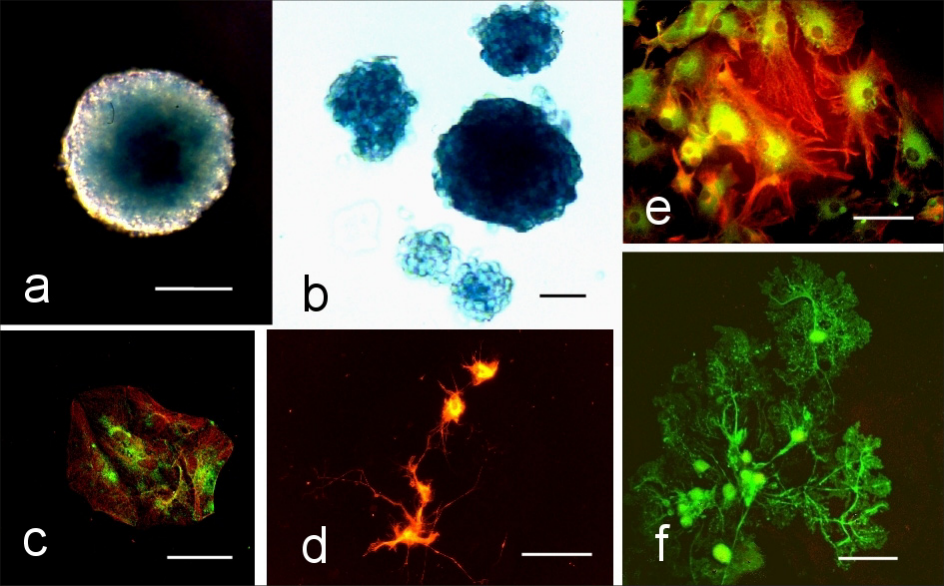
Neurosphere stem cell lineages express PTPσ. a) A neurosphere derived from the SVZ of a PTPσ(+/-) mouse brain and reacted for the histochemical localization of β-galactosidase. Nomarski phase contrast inverted microscopy. Scale bar = 50 μm. b) Histochemical localization of the PTPσ reporter gene β-galactosidase in neurospheres originating from a PTPσ(-/-) mouse. Phase contrast inverted microscopy. Scale bar = 25 μm. c) Co-expression of the progenitor cell marker NG2 (green) and the neuronal lineage marker tubulin (red) in cells differentiated from a PTPσ(-/-) mouse. Epi-fluorescence microscopy. Scale bar = 50 μm. d) Differentiated neurons derived from a PTPσ(-/-) neurosphere. The merged image shows the co-expression (yellow) of β-galactosidase and β-III tubulin in the cytoplasm. Epi-fluorescence microscopy. Scale bar = 50 μm. e) Astrocytes contain GFAP (rhodamine-red) and β-galactosidase (fluorescein-green) when differentiated from a PTPσ(-/-) neurosphere. Epi-fluorescence microscopy. Scale bar = 50 μm. f) Mature oligodendrocytes derived from a PTPσ(-/-) mouse stained with the 04 antibody. Epi-fluorescence microscopy. Scale bar = 50 μm.

To determine whether PTPσ is expressed in the neuronal stem cells, β-galactosidase expression, included in the knockout cassette and driven by the endogenous *Ptprs *promoter, was analyzed. As seen in Figure 2, the neurospheres from the PTPσ (-/-) and the (+/-) mice exhibited β-galactosidase expression (reporting *Ptprs *expression), demonstrating that PTPσ is normally present in these neural stem cells. Neurospheres produced from the wildtype animals showed no β-galactosidase staining, as expected. The neurospheres generated from all 3 genotypes consisted of a heterogeneous makeup of progenitor cells and lineage committed cells. The spheres often contained cells with the NG2 cell progenitor marker and cells co-expressing GFAP or tubulin. When the neurospheres were induced to differentiate, we observed differentiated neurons, astrocytes and oligodendrocytes that co-expressed β-galactosidase with the appropriate end cell marker (Figure 2). There was no apparent difference between the different genotypes in the capacity for neurospheres to generate the 3 separate cell lineages, nor was there any tendency for a genotype to produce a particular lineage.

### Neurons lacking PTPσ show altered migration patterns and enhanced neurite outgrowth

**Figure 3:**
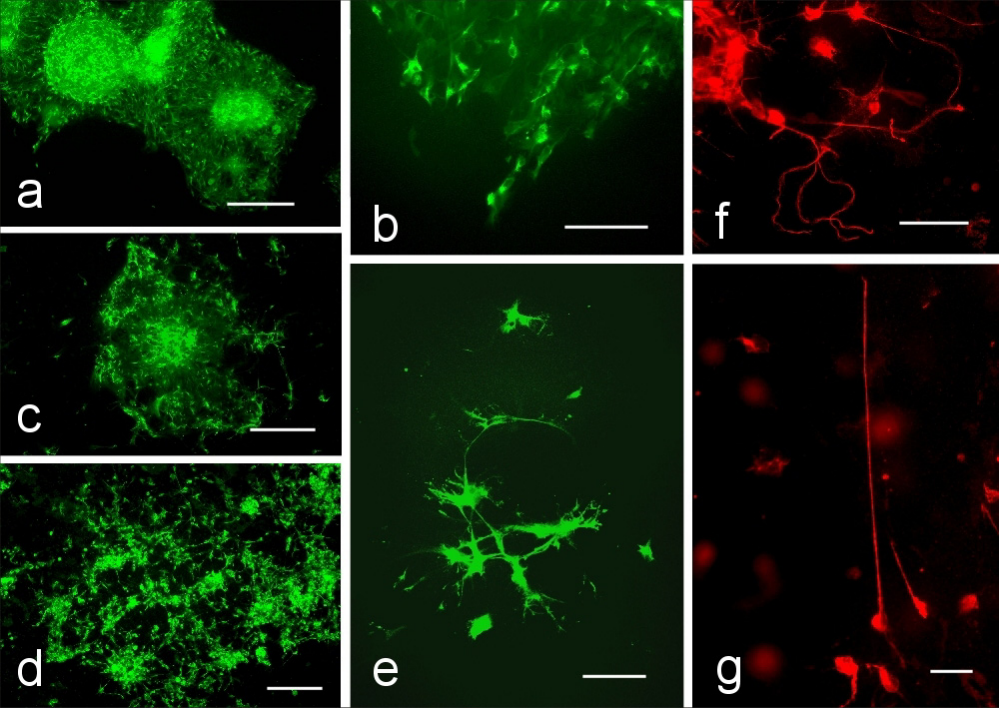
Neurons show enhanced migration patterns and neurite outgrowth when differentiating from neurospheres derived from PTPσ (-/-) mice. All images taken after 7 days of differentiation. Epi-fluorescence with the β-III tubulin antibody. A. Morphological appearance: a) Appearance of neurospheres from PTPσ(+/+) mice grown on laminin coated coverslips. Scale bar = 150 μm. b) Higher magnification of (a) to show neuronal progeny derived from the PTPσ(+/+) genotype. Scale bar = 100 μm c) Appearance of the neuronal migration from PTPσ(+/-) derived neurospheres. Scale Bar = 100 μm d) Neurons that differentiate from neurospheres from a PTPσ(-/-) mouse appear extensively distanced from the neurosphere of origin. Scale bar = 100μm. e) Neuronal differentiation from a PTPσ(+/-) neurosphere. Scale bar = 50μm. f) Long random processes characterize PTPσ(-/-) neurons. β-III tubulin immunocytochemistry. Scale bar = 50μm. g) High power view of a long process from a neuron derived from a PTPσ(-/-) neurosphere. β-III tubulin immunocytochemistry. Scale bar = 25μm.

**Figure 4:**
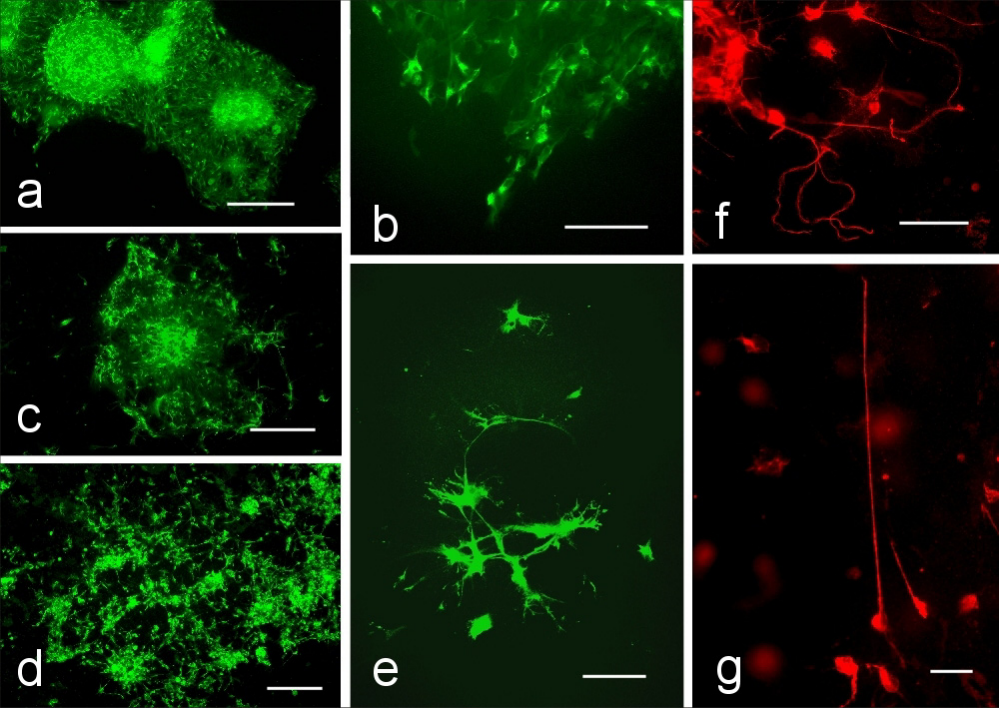
Neurite length of differentiated neurons (identified by b-III tubulin immunocytochemistry) grown on laminin coated glass coverslips. The data (average of all neurites ±SEM) show a significant increase in neurite outgrowth from the PTPs(-/-) mice (KO) relative to sibling (Sib) controls. Data represent 4 separate experiments with a total of 469 neurites measured for the knockout mice and 573 neurites for the siblings. KO represents mice genotyped as PTPs (-/-) and Sib represents mice genotyped as PTPs(+/-) or PTPs (+/+).  There was no difference in the length of neurites between the neurons from the PTPs(+/+) or (+/-) neurospheres, hence their data were combined.

The neuronal lineage derived from PTPσ (-/-) mice demonstrated a different migration pattern compared to both heterozygotes and wild type mice (Figure 3). Neurons generated from the PTPσ (-/-) neurospheres migrated over large areas of the laminin substrate. These neurons, identified by β III-tubulin expression, moved long distances from the center of the neurospheres to often create overlapping zones of migration between adjacent neurospheres. The neurons from the PTPσ (+/-) and (+/+) mice remained close to the neurosphere of origin and were observed in closely associated groups. Processes derived from PTPσ (-/-) neurons were significantly (p <0.001) longer than the neurons from heterozygote PTPσ (+/-) and wildtype neurons. The PTPσ (-/-) neurons tended to have one neurite that was considerably much longer than the other neurites. Seven days after the induction of differentiation, the PTPσ (-/-) neurons had average neurite lengths of 77.0 ± 2.2 μm, whereas the PTPσ(+/-) and wildtype neurons showed neurite lengths of 34.0 ± 16.6 μm (Figure 4). No differences in the migration patterns of astrocytes and oligodendrocytes derived from the PTPσ (-/-) or sibling controls were observed. These cells remained close to the neurosphere of origin.

## Discussion

We have demonstrated the expression of PTPσ in neural stem cells and in each of the 3 main lineages derived from the neural stem cell, as well as a role for this gene in neuronal lineage differentiation. To our knowledge, this is the first direct demonstration of PTP expression in neural stem cells isolated from the SVZ, in line with our earlier observation of strong expression of PTPσ in the SVZ [22]. Moreover, it is also the first demonstration of a role for a PTP (PTPσ) specifically in the growth of stem cell-derived neurons, but not in the development of the other lineages (astrocytes and oligodendrocytes) under our culture conditions. Identification of glial substrates for this gene may offer insight into specific function(s) for astrocytes and oligodendrocytes. Bernabeu and coworkers have recently demonstrated the expression of LAR in progenitor cells of the dentate gyrus within the hippocampus of mice, and showed increased neurogenesis in LAR deficient dentate gyrus [26]. This is interesting given the previously documented stimulatory role of LAR in neuronal growth (see below), including hippocampal cholinergic neurons [13,14,33].

The *Ptprs *gene does not appear to influence the formation and cellular makeup of the neurosphere. However, while we did not observe any significant phenotypic differences in the astrocyte or oligodendrocyte cell lineages produced by the neurospheres derived from the PTPσ deficient mice relative to wild type mice, prominent phenotypic differences were noted in the neuronal derivatives of these neurospheres. In particular, neurite outgrowth was accelerated in the knockout animals, suggesting that PTPσ provides inhibitory stop signals for neurite growth.

A number of receptor PTPs, particularly from the LAR or LAR-related families, have demonstrated an ability to promote neurite or axonal outgrowth *in vitro*. Examples include LAR [27], PTPδ [28,29], PTPμ[30] and PTPk [31]. Regulation of neurite outgrowth by LAR has also been shown *in vivo *[22,32,33]. Our results suggest that unlike these PTPs, PTPσ, which is highly homologous to LAR and PTPδ, acts as an inhibitor of neurite outgrowth. This supports our previous studies documenting increased regeneration rate of the sciatic nerve in PTPσ deficient mice following injury *in vivo *[23], an observation subsequently confirmed by studies of facial nerve regeneration carried out by Thompson et al [24]. Together with our present data, these findings support the notion of a role for PTPσ in inhibiting neurite outgrowth. This is in contrast to the finding that sciatic nerve crush in LAR knockout mice resulted in delayed nerve regeneration [34], suggesting that LAR (unlike its close relative PTPσ) promotes nerve regeneration and growth *in vivo*. McLean and colleagues [23] also documented increased errors in axonal pathfinding in PTPσ knockout mice, complementing the invertebrate studies with *Drosophila *DLAR [35].

In our study, differences in the neuronal migratory patterns from the neurospheres existed between PTPσ deficient mice and their sibling matched controls when differentiated on laminin coated glass coverslips. The intracellular pathway(s) regulating PTPσ mediated control of neurite growth in the CNS and PNS are unknown since the substrate(s) for this phosphatase has/have not been described. Other receptor PTPs (eg PTPμ), related to PTPσ, have been reported to regulate the cadherin-catenin complex. Our preliminary examination of the neurite lengths of neurons grown on the N-cadherin substrate did not reveal any significant differences in neurite length between the PTPσ (-/-) mice and the siblings under our current assay conditions. These preliminary data suggest that neurite growth regulated by PTPσ is also dependent on extracellular substrata/factors, likely in addition to intrinsic factors.

Our studies corroborate the growing body of evidence illustrating a very diverse make-up to the developing neurosphere [36,37]. The intrinsic complexity of the various developmental stages of neural lineages was apparent when staining with the different combinations of precursor and lineage specific antibodies. Our understanding of antibodies thought to identify exclusive steps in precursor cell development, glial or neuronal lineages is constantly changing. The NG2 antibody, thought to identify a chondrotin sulfate proteoglycan on the surface of glial cells only, co-existed with cells expressing neuronal markers in our neurospheres. This same antibody has been used by Belachew and colleagues [38] to reveal similar results *in vivo*, including the generation of neurons from NG2 positive glial precursors.

## Conclusion

In conclusion, we have performed adult stem cell isolation procedures in mice lacking PTPσ. The combination of these techniques provides a unique and powerful opportunity to study the role of single genes on the developmental aspects of stem cell derived glial and neuronal lineages. Our results support a role for PTPσ in specifically suppressing the neurite outgrowth from stem cell derived neurons (but not other lineages) and modulating their migration patterns.

## Methods

All the experiments with the mice followed the guidelines set out by the Canadian Council on Animal Care, and they were approved by the Animal Research Ethics Board of McMaster University.

### Animals and genotyping

The PTPσ knockout mice were generated and characterised as described previously [20]. The knockout cassette used to generate the mice included the β-galactosidase gene, to allow detection of expression by lacZ staining. PTPσ (+/-) mice were phenotypically indistinguishable from wild type animals. Three cohorts of PTPσ (-/-) could be identified: 60% of PTPσ (-/-) animals die within 2 days after birth, likely from hypoglycemia [25]. Approximately 38% live until two to three weeks of age and then succumb to a wasting syndrome. Only 2.5% of PTPσ (-/-) mice survive to adulthood, are approximately 25% – 50% smaller by weight, but are fertile.

### Isolation of neural stem cells and neurosphere expansion

Stem cell isolation procedures were adapted primarily from Weiss and colleagues [39,40]. The brains from the PTPσ (-/-), (+/-) and (+/+) mice, aged 1 to 4 weeks were removed and suspended in artificial cerebrospinal fluid (aCSF) containing 0.012 M NaCl, 0.005 M KCl, 0.03 M MgCl_2_, 0.026M NaHCO_3_, 0.01 M glucose and 0.097 mM CaCl_2 _in dH_2_O. The brains were bisected along the sagittal plane to separate the hemispheres and permit isolation of a thin tissue layer outside the ventricles containing the SVZ. The isolated tissue was dissociated with a mixture of enzymes (1.0 ml of aCSF containing; 0.13 mg kynurenic acid (4-hydroxyquinoline-2-carboxylic acid, Sigma, St. Louis, MO), 0.66 mg type 1-S hyaluronidase (Sigma, St. Louis, MO) and 1.3 mg trypsin (Sigma, St. Louis, MO) for 90 min in a shaking water bath set to 35°C. The sample was triturated with a Pasteur pipette thirty times every 15 min. The single cell suspension was re-suspended in 4.0 ml of serum free media containing 1.6 μl of ovomucoid (trypsin inhibitor, Sigma, St. Louis, MO). The serum free media (SFM) consisted of DMEM/F12 (Life Technologies, Burlington ON) with 0.03M glucose, 0.005 M hepes buffer (Sigma St. Louis, MO), 20 nM progesterone (4-pregnene-3,20-dione, Sigma, St. Louis, MO), 60μM putrescine (Sigma^R^St. Louis, MO. 1,4-diaminobutane; tetra-methylenediamine), 0.1 mL insulin-transferrin-sodium selenite (Roche), 2.0 mL B27 Growth supplement (Life Technologies, Burlington ON), 20 μL epidermal growth factor (20 ng/ml, Sigma, St. Louis, MO), 20 μl basic fibroblast growth factor (10 ng/ml, Sigma, St. Louis, MO) and 7.32 μl heparin (Sigma, St. Louis, MO) in a total of 100 mls of SFM. The cells were centrifuged at 250 g for 3 min, placed into fresh SFM and plated at a density of 10 viable cells per μL in serum free media to achieve clonally derived neurospheres [41]. Each well of the 24 well dishes received 500 μL of the cell suspension. The plates were maintained in a humidified 5% CO_2_/95% air incubator at 37°C. Developing neurospheres were prominent after 3–7 days in culture and were passaged on a weekly basis. For the differentiation studies, the neurospheres were used at the second or the third passage.

To enhance the generation of oligodendrocytes from the neurospheres, a slightly modified medium was used [42]. An oligodendrocyte specification medium (OSM) consisted of 100 ml of DMEM/F12 with 0.5 mg insulin, 1.61 mg putrescine, 5.0 mg transferrin, 0.46 gm D(+) galactose (0.025 M) and 200 μL of insulin-transferrin-sodium selenite. The OSM was used in combination with the SFM at a ratio of 5.3 ml to 6.7 ml (respectively) to make 12.0 ml of media.

### Immunocytochemistry

Neurospheres were washed in PBS, fixed in 4.0% buffered paraformaldehyde for 5 minutes then washed twice with PBS prior to the incubation with the primary antibodies. Progenitor cells were detected by nestin expression (Pharmingen), NG2 (Chemicon) and the EGF-receptor (Santa Cruz Biotechnology, Santa Cruz, CA). Differentiated cells were assessed for their immunoreactivity towards antibodies specific for β III-tubulin or PGP 9.5 (Sigma), glial fibrillary acidic protein (GFAP) (Dako) and O4 (Chemicon) to detect the presence of neurons, astrocytes and oligodendrocytes, respectively. All double labels were conducted using primary and secondary antibodies applied independently over two consecutive days. The appropriate species specific secondary antibody linked to fluorescein or rhodamine was applied and the spheres were viewed by confocal or epifluorescence microscopy. Controls involved the same procedures with the omission of the primary antibody.

### Detection of the β-galactosidase reporter gene

Spheres were fixed as described above and re-suspended in 1.5 ml of: 1.0 mg/ml 5-bromo-4-chloro-3-indoyl-β-D-galactopyranoside (Roche), 5.0 mM potassium ferrocyanide, 5.0 mM potassium ferricyanide and 2.0 mM magnesium chloride (in PBS). Cells were placed in a standard humidified 5% CO_2_/95% air incubator at 37°C for 5 to 15 minutes until the blue color was visible when observing the spheres under a dissection microscope. In addition, some of the cells induced to differentiate were processed for fluorescence immunocytochemistry using a primary antibody to β-galactosidase (Sigma, St.Louis, MO).

### Measurement of neurite length

The extent of neurite outgrowth from the neuronal progeny generated from neurospheres was determined on laminin coated glass coverslips. Circular coverslips (Bell) were immersed in 95% ethanol, passed over a bunsen burner flame and placed into each well of a 24 well plate. Laminin (12 μl, SIGMA) was diluted in 12.0 mL of SFM and used to cover the surface of the coverslips. The 24 well plates were placed in a humidified 5%CO_2_/95% air incubator at 37°C for 45 minutes. The laminin/media was removed and replaced with the neurosphere suspension in SFM. To induce differentiation of the neurospheres, the mitogens were withdrawn from the media and two drops of media containing 10% fetal bovine serum in DMEM was added to each well [43]. An alternative method to induce differentiation involved reducing the growth factors to 50% and adding the media containing the fetal bovine serum. After 7 days in culture, the cells were processed for β III-tubulin immunocytochemistry. Digital images of the neurons were captured and the processes measured using the LSM 5 image browser software or Axiovision 4.5 software (Zeiss). Only processes that had a clear origin from the cell body were measured. All statistical analysis was performed with SPSS or MiniTab software.

## Authors' contributions

DLK, LKKP and MMA isolated stem cells, expanded neurospheres and performed immunocytochemistry. DLK and LKKP analyzed the neurite lengths. MMA carried out the oligodendrocyte enrichment and immunocytochemistry. RS performed substrate purifications and genotyping. DR and LCD conceived the study, coordinated experiments and drafted the manuscript.

## Figures and Tables

**Table 1:** Number of neurospheres generated/well (n = 9) after 7 days in culture. Average +/- SE. ANOVA indicated no significant difference between the different genotypes.

Genotype	EGF	bFGF	EGF & bFGF
**-/-**	50.3 +/- 14.14	43.3 +/- 21.18	108 +/- 9.84
**+/-**	60.3 +/- 14.78	39.6 +/- 12.45	130 +/- 7.51
**+/+**	31.3 +/- 16.34	34.0 +/- 10.07	84.3 +/- 28.16

**Table 2:** Cell marker combinations used to identify cell lineage heterogeneity in the neurospheres. (+) indicates detection of the antibody. (-) indicates that the antibody combination was not co-expressed in cells within the neurospheres.

Marker Combination	Double labelled cells in neurospheres	Single labelled cells in neurosphere
04 plus GFAP	+	+
04 plus PGP	-	+
04 plus NG2	+	+
tubulin plus GFAP	-	+
tubulin plus NG2	+	+
NG2 and GFAP	+	+
